# Chemotherapy Curability in Leukemia, Lymphoma, Germ Cell Tumors and Gestational Malignancies: A Reflection of the Unique Physiology of Their Cells of Origin

**DOI:** 10.3389/fgene.2020.00426

**Published:** 2020-06-09

**Authors:** Philip Savage

**Affiliations:** Department of Oncology, Brighton and Sussex University Hospitals, Brighton, United Kingdom

**Keywords:** cancer, chemotherapy, cure, phenotype, apoptosis

## Abstract

Cytotoxic DNA damaging chemotherapy brings clinical benefits in the treatment of many metastatic malignancies. However routine curative treatment remains restricted to a small number of malignancies including acute leukemia, high grade lymphoma, germ cell tumors, gestational malignancies and some of the rare childhood cancers. The detailed explanation for this dramatic divergence in outcomes remains to be elucidated. However, we have previously argued that there is a strong correlation between presence of the unique genetic events of immunoglobulin gene variable/diversity/joining (VDJ) recombination, somatic hypermutation (SHM), meiosis, nuclear fusion and gastrulation occurring in cells of origin of these malignancies and their high sensitivity to DNA damaging chemotherapy. In this study we have reviewed some of the basic physiological information relating to the specialized activity and sensitivity to DNA damage mediated apoptosis of normal cells undergoing these processes. In each of unique genetic events there are dramatic changes in apoptotic sensitivity. In VDJ recombination and somatic hypermutation over 95% of the cells involved undergo apoptosis, whilst in meiosis and nuclear fusion there are dramatic short term increases in the apoptotic sensitivity to DNA damage. It is apparent that each of the malignancies arising during these processes retains some of the unique phenotype associated with it. The impact of the physiological differences is most clearly seen in the two non-mutational malignancies. Gestational choriocarcinoma which arises shortly after nuclear fusion is routinely curable with chemotherapy whilst CIMP-positive ependymomas which is not linked to any of the unique genetic events is highly resistant. A similar pattern is found in a pair of malignancies driven by a single driver mutation. Infantile acute lymphoblastic leukemia (ALL) arises in a cell undergoing the early stages of VDJ recombination and has a 40% cure rate in contrast pediatric rhabdoid malignancy which is not linked to a unique genetic event responds very poorly to chemotherapy treatment. The physiological changes occurring in cancer cells at the time of the malignant transformation appear to have a major impact on the subsequent sensitivity to chemotherapy and curability. New therapies that impact on these pathways may be of therapeutic value.

## Introduction and Hypothesis

The additional genetic changes that can occur in tumors after the development of malignancy have been extensively studied and considerable data indicates that tumor genetic heterogeneity is an important factor in resistance to many cancer therapies (Gerlinger et al., 2012; Alizadeh et al., 2015).

Whilst the majority of metastatic malignancies remain resistant to curative drug treatment, a number of rarer malignancies, comprising gestational choriocarcinoma, testicular and ovarian germ cell tumors, acute leukemia, high grade lymphoma, Hodgkin’s disease and some of the rare childhood malignancies have been routinely curable with cytotoxic chemotherapy drugs for more than 60 years (Hertz et al., 1956; Chabner and Roberts, 2005; DeVita and Chu, 2008).

The explanation for this dramatic divergence in curability between these relatively rare malignancies and the more common incurable metastatic malignancies has long been a subject of great interest and scientific debate (Savage et al., 2009; Pritchard et al., 2013; Housman et al., 2014; Romano et al., 2016).

**FIGURE 1:**
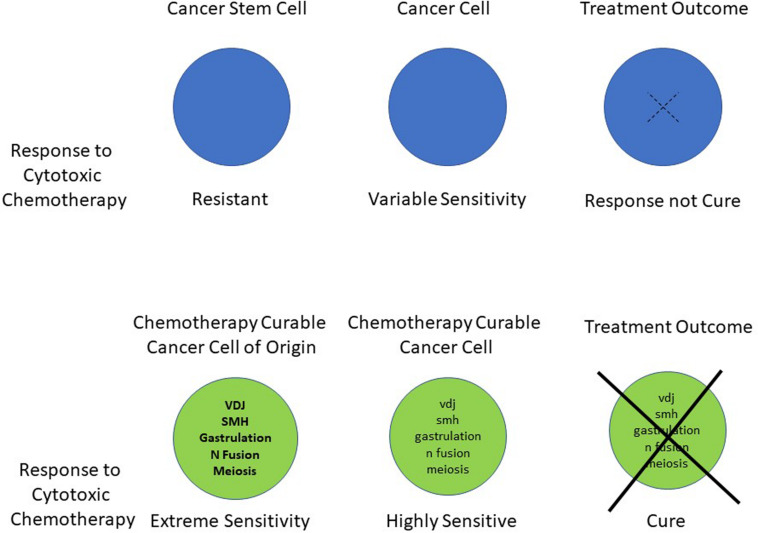
Schematic summary of the hypothesis indicating the unique genetic processes, which are associated with dramatic changes in apoptotic sensitivity. These occur in the cancer cells of origin and a remnant of this activity persists in their subsequent malignant cells. This ongoing phenotype leads to a heightened apoptotic response to DNA damaging chemotherapy.

Historically the rate of cell division and the development of mutations within the tumor have been regarded as two of the key features in determining sensitivity and resistance to cytotoxic chemotherapy (Dean et al., 2005; Mitchison, 2012; Holohan et al., 2013). More recently we have suggested that a further central component determines heightened chemotherapy sensitivity and cancer curability. The overview of the hypothesis is shown in Figure 1. This shows the persistence in the malignant cells of at least part of the unique phenotype that their transient cells of origin hold at the time of their malignant transformation (Savage, 2015). The result of this is to produce extreme sensitivity to DNA damaging chemotherapy and radiation in the resultant malignancies.

Whilst the genetic event of mitosis is common to all human cells, the chemotherapy curable malignancies each appear to have a very close temporal association between their cell of origin and the physiological specialized unique genetic events. As shown in more detail in Table 1 one of the events of nuclear fusion, immunoglobulin VDJ gene recombination, immunoglobulin gene somatic hypermutation, meiosis or gastrulation is closely linked to the cell of origin of the each of the chemotherapy curable malignancies (Savage, 2015).

**TABLE 1:** Comparison of the chemotherapy curability for varying malignancies and their relationship with the unique genetic events.

Malignancy	Cell of Origin	Genetic Event	Chemotherapy Cure Rate
ALL	Pro B cell	Immunoglobulin Gene VDJ Rearrangement	90% children (Pui et al., 2014) 30% adults (Sive et al., 2012)
DLBCL Hodgkin Lymphoma	Germinal Center B Cell	Immunoglobulin Gene Somatic Hypermutation/Class Switching	DLBCL 60% (Fu et al., 2008) Hodgkin Lymphoma 85% (Canellos et al., 1992)
Gestational Trophoblastic Tumors	Trophoblast cell (early pregnancy cell)	Nuclear fusion	Post molar pregnancy 100% (Sita-Lumsden et al., 2012) Choriocarcinoma 95% (Alifrangis et al., 2013) PSTT 50% (Schmid et al., 2009)
Germ Cell Tumors	Arrested Gonocyte	Meiosis	Testicular cancer 75% (Hanna and Einhorn, 2014) OGCT 80% (Murugaesu et al., 2006)
Childhood Malignancies	Unknown	Gastrulation	Ewing Sarcoma 74% (Smith et al., 2010) Wilms Tumor 90% (Smith et al., 2010)
Mantle Cell Lymphoma CLL Myeloma	B cell (non-VDJ or SHM)	None	0% (Greipp et al., 2005; Schulz et al., 2007; Pflug et al., 2014)
Common epithelial malignancies	Epithelial Cell/Stem Cell	None	0% (Chabner and Roberts, 2005)

There is increasing data to support the hypothesis that these malignant cells, retain components of the normally transient physiological changes occurring in their parent cells. As we will discuss in this review, it is apparent that the cells undergoing these unique genetic events transiently gain extremely high sensitivity to DNA damage mediated apoptosis. Retention of these profiles within the malignant cells appears to lead to heightened sensitivity to DNA damage induced apoptosis and hence curative cytotoxic chemotherapy treatment.

## Cell of Origin, Malignant Transformation and Frozen Development

The concept that a malignant cell retains much of the characteristics of the cell of origin from which it has arisen is well established and the impact of this is most clearly illustrated in the lymphoid malignancies (Küppers et al., 1999). The most apparent feature of this developmental freezing in B cell malignancies is the static morphology and fixed antigenic markers that define the appearance and cell surface marker phenotype of the differing B cell malignancies (Shaffer et al., 2002).

As can be seen in Table 2, it is apparent that the malignancies arising along the B cell developmental pathway include a wide range of differing cell types and physiologies. These include acute undifferentiated leukemia, acute lymphoblastic leukemia, mantle cell lymphoma, diffuse large B cell lymphoma, Hodgkin’s lymphoma, chronic lymphocytic leukemia and myeloma. Each of these malignancies has very differing morphology, cell surface antigens, typical mutational rates, physiological characteristics and cytotoxic chemotherapy cure rates despite their common B cell ancestry. In addition to the fixed antigenic phenotype corresponding to the cell of origin, the malignant B cells also retain gene expression profiles that are very similar to that of the corresponding normal B cell parent (Andersson et al., 2005, 2010).

**TABLE 2:** Comparison of the cell of origin, mutational burden, genetic activity and chemotherapy cure rate for selected B cell malignancies.

Malignancy	Cell of Origin	Tumor Mutation Burden (Mutations per Megabase) (Chalmers et al., 2017)	Genetic Activity	Chemotherapy Cure Rate
AUL	HSC/LSC	Unknown	VDJ±	20% (Heesch et al., 2013)
Pediatric ALL	Fetal liver HSC	1 mutation	VDJ±	40% (Pieters et al., 2007)
B ALL	Pro B cell	1.7	VDJ+++	90% (Pui et al., 2014)
CLL	Pre-Germinal Center B Cell	1.7	Nil	0% (Pflug et al., 2014)
Mantle Cell NHL	Pre-Germinal Center B Cell	3.3	Nil	0% (Schulz et al., 2007)
DLBCL	Germinal Center	10.0	SHM+++	65% (Canellos et al., 1992)
Follicular NHL	Memory B cell	8.3	SHM+	0% (Schaaf et al., 2012)
Lymphoplasmacytic lymphoma	Memory B cell	No data	Nil	0% (Dimopoulos et al., 2014)
Myeloma	Plasma Cell	2.2	Nil	0% (Greipp et al., 2005)

*Data on the mutational burden is taken from Chalmers et al. (2017).*

A parallel pattern of frozen development is also seen in other malignancies that have linear developmental pathways. In the rarer diagnoses of T cell malignancies, acute T cell leukemia arise from cells at the earliest developmental point from the hematopoietic stem cell (De Bie et al., 2018), whilst the more indolent malignancies of *Sezary* syndrome and mycoses fungoides arise from mature effector T cells (Campbell et al., 2010).

Similarly, in the gestational trophoblastic malignancies, choriocarcinoma retains the phenotypic and methylation characteristics of a very early trophoblast cell (Mao et al., 2007; Savage et al., 2019). Whilst the less chemotherapy sensitive rarer malignancies of placental site trophoblastic tumor (PSTT) and epithelioid trophoblast tumor arise from more developmentally mature cells (Kurman et al., 1984).

## Unique Genetic Events, Natural Physiological Changes, Impact on Apoptotic Sensitivity and Chemotherapy Curability

### Acute B Cell Leukemia and VDJ Recombination

During the development pathway of normal B cells, the inherent sensitivity of the transient cells and their related malignancies to the induction of apoptosis via DNA damage varies dramatically. Within a short period of time developing B cells move from hematopoietic stem cells, which are inherently very resistant to DNA damage mediated apoptosis (Mohrin et al., 2010; Biechonski et al., 2018) to pro-B cells that can give rise to B-ALL.

The process of VDJ recombination of the immunoglobulin genes is the key defining feature of the early development phase of B cells and is the initial mechanism that allows the production of the width of antibody response from the limited pool of germ line immunoglobulin genes (Tonegawa, 1983).

The VDJ recombination process includes the cutting and re-joining of the immunoglobulin genes in a process involving the VDJ recombinase system (Oettinger et al., 1990). Within this process, the expression and activation of the key RAG1 and RAG2 enzymes is tightly controlled, occurring at significant levels only in B and T cells and is restricted to just a brief time in their overall cellular development pathway (Kuo and Schlissel, 2009).

The initiation of the VDJ phenotype and end of the VDJ process occur as a result of epigenetic changes very early in B cell and T cell development. The key components of the VDJ process, including the expression of RAG1, RAG2, DNTT (TdT) and ADA, are switched on early as the cells move from hemopoietic stem cell to common lymphocyte progenitor (CLP) and then are increased in width and intensity as cells move through the pro-B cell stage (Hystad et al., 2007).

Alongside the changes in gene expression there are also changes in the physical structure of the DNA encoding the immunoglobulin genes and their recognition sequences. These changes occur by alterations in the placement of nucleosomes that produce enhancement to the accessibility of the RAG recombinase to the immunoglobulin genes (Pulivarthy et al., 2016). These processes combine to focus VDJ activity predominantly to the immunoglobulin genes, although it is apparent that the process still retains significant risk of off target mutation and adverse oncogenic outcome (Tsujimoto et al., 1985; Schlissel et al., 2006).

In normal B cell development, the activity associated with the VDJ phenotype is then lost as the VDJ recombinase system is switched off as the cells move through to the stage of the immature B cell (Llorian et al., 2007). During the process of VDJ recombination, the B cell phenotype is characterized by an acquired balance between enhanced apoptotic pressures and competing survival signals. It is estimated, in murine studies, that cells undergoing VDJ recombination are dividing every 16 h (Opstelten and Osmond, 1983) and that 97% of these B cells die an apoptotic death at this stage (Liu et al., 1989; Osmond, 1991).

B-ALL is characteristically associated with a number of key mutations, including PAX5, IKZF1, TCF3 and EBF1. These mutations impact on the activity of genes associated with the regulation of normal B cell development (Inaba et al., 2013). Disruption of the activity of these genes can lead to a block in B cell differentiation and maturation and result in developmental arrest (Liu et al., 2014). The impact of this is to prevent the B cell either dying an apoptotic death or successfully maturing and moving through to the next stage in development.

There is appreciable data to indicate that the B cell malignancies arising at this part in their development also retain an ongoing degree of activity of the VDJ phenotype. A number of studies have reported that in B-ALL there is detectable ongoing activity of the VDJ system in the malignant cells (Bird et al., 1988; Yoneda et al., 1993; Beishuizen et al., 1994; Li et al., 2004; Gawad et al., 2012). As a result, it appears the pro B cell as it becomes frozen in its developmental phenotype by the onset of malignancy retains at some of the activity of the VDJ phenotype and its associated mechanisms including heighten apoptotic sensitivity.

#### Physiological Changes in Apoptotic Sensitivity During VDJ Recombination

There is a large amount of clinical data from the 1940s onward documenting the extreme sensitivity (Farber and Diamond, 1948) and subsequent routine curability for B-ALL to DNA damaging cytotoxic chemotherapy (Pinkel, 1971). However, there is relatively little work exploring the sensitivity to the induction of apoptosis by DNA damage in the cell of origin.

Whilst hemopoietic stem cells appear able to tolerate DNA damage and have a high threshold for the induction of apoptosis (Durdik et al., 2017) developing lymphocytes vary significantly in their sensitivity to DNA damage during the B cell development pathway.

**FIGURE 2:**
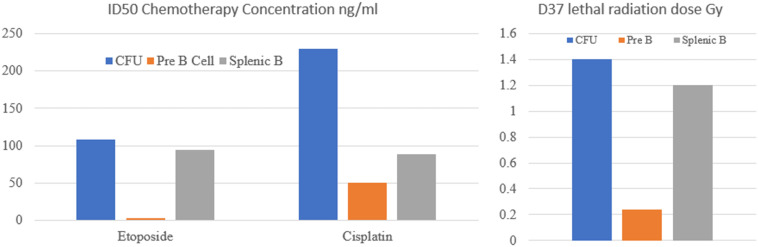
Sensitivity of colony forming units (CFU), pre-B cells and mature splenic B cells to cytotoxic chemotherapy drugs and radiation. ID50 is the drug concentration required to kill 50% of cells. D37 is the radiation dose (Gy) required to kill 37% of cells. It is apparent that Pre B cells are significantly more sensitive to DNA damaging agents than CFU or splenic B cells.

An insight into the dramatic changes in the apoptotic threshold to DNA damage during B cell development is seen in a paper published in 1993 by Griffiths et al. (1994a). In this study the authors compared the *in vitro* sensitivity of IL-7 dependent B cells, which equate to pro-B cells, and that of other earlier and later developmental lymphoid lines to cytotoxic chemotherapy drugs. As shown in Figure 2 the cells at these differing developmental stages had markedly differing sensitivity to chemotherapy drugs. This is most clearly shown with etoposide where the 50% inhibitory dose (ID50) dose varies from 108 ng/ml for CFU, falls to 3.2 ng/ml for pre-B cells and then rises to 95 ng/ml for mature B cells (Griffiths et al., 1994a).

A parallel study examined the sensitivity of these B cells to radiotherapy also showed a similar pattern. The lethal radiation dose permitting 37% survival (D37) was 1.4Gy for the CFU, 0.24Gy for the pre-B cells and 1.2Gy for mature B cells (Griffiths et al., 1994b). Similar results were also reported examining the radio sensitivity of B cell lines of different developmental stages. The greatest sensitivity was seen in the pro B cell lines with greater resistance seen in both lymphocyte progenitor cells and also in B cells that have completed VDJ recombination (Uckun et al., 1991).

#### The Impact of the Timing of Oncogenesis on B ALL Chemotherapy Sensitivity Characteristics

Within the overall diagnosis of B-ALL, a number of key sub-types arise at slightly differing points in the early stages of B cell development. These malignancies have significantly differing clinical characteristics and also differing outcomes to cytotoxic chemotherapy treatment. The relationship of these diagnoses and their chemotherapy sensitivity appears to have a potential relationship to the developmental timing and the activity of the VDJ system at the time of malignant transformation.

Malignant transformation occurring very early in the development of the B cell pathway prior to the point at which B-ALL would arise can lead to the development of two relatively rare forms of leukemia, infantile ALL and acute undifferentiated leukemia.

The rare diagnosis of infantile ALL arises from the stem/progenitor cells present in the fetal liver (Agraz-Doblas et al., 2019) and has a chemotherapy cure rate of approximately 40% (Pieters et al., 2007). This malignancy is unusual in that it generally occurs as a result of a single mutation in the mixed lineage leukemia (MLL) gene (Biondi et al., 2000). Aside from this mutation the rest of the malignant cell’s genome appears otherwise mutationally bland (Dobbins et al., 2013; Andersson et al., 2015).

Review of the activity of the VDJ system in *t*(4;11) infant acute lymphoblastic leukemia indicates that whilst there is generally expression of TdT, a key enzyme in the VDJ system (Liu et al., 2004) but there is a low frequency of completed immunoglobulin and T-cell receptor gene rearrangements (Peham et al., 2002). As a result, it appears that this malignancy is likely to arise during the very earliest phase of VDJ recombination, when the VDJ phenotype associated apoptotic sensitivity is still evolving.

Acute undifferentiated leukemia is also rare and is characterized by an absence of lymphoid or myeloid lineage-specific antigens. Overall early genetic assessment of acute undifferentiated leukemia indicates a malignancy characterized by a stem-cell-driven gene expression pattern that lacks any of the common recurrent genotype aberrations (Heesch et al., 2013). More recent information indicates that mutations of genes linked to lymphocyte development are common and that there is expression of TdT, an early component of the VDJ process (Weinberg et al., 2019).

Clinically this malignancy carries a much poorer response to treatment and lower cure rate than the more frequent cases of B-ALL, with a reported cure rate in the region of 20% (Heesch et al., 2013). Genetic assessment of the VDJ status of this malignancy indicates that acute undifferentiated leukemia cells do not have clonal rearrangements of the B cell receptor indicating that these malignant cells arise prior to the commencement of effective VDJ recombination (Lao et al., 2019).

Conventional B-ALL in children has high cure rate approaching 90% (Pui et al., 2014) however in adults the cure rates are much lower, and the overall biology appears significantly different (Rowe, 2010). There are a number of possible explanations for this divergence in outcome including better tolerability of intensive chemotherapy in younger patients and the high prevalence of adverse prognostic factors in older patients. However, it is possible that differences in the underlying reduced intensity of the activity of the VDJ system in adults compared to children may also play an important role (Jensen et al., 2013a, b).

Whilst the full mechanisms for the enhanced sensitivity to chemotherapy treatment tracking in parallel with the VDJ phenotype is not yet detailed, it appears to be a strong association. In contrast the relationship between curability and the mutational load of these differing types of B cell malignancies appears much less strong as shown in Table 2.

#### Post VDJ Pre Somatic Hypermutation B Cell Malignancies

After the VDJ process is completed, the healthy B cell that has successfully recombined its immunoglobulin genes moves from a pro-B cell to an immature B cell. The VDJ process is completed and the phenotype moves away from the previously seen finely balanced apoptosis/survival pressures.

At this point in B cell development if malignancy occurs the resultant diagnosis can include mantle cell lymphoma or CLL (without hypermutated antibody variable regions) each of these whilst responsive to chemotherapy are not currently curable (Crespo et al., 2003; Fernàndez et al., 2010).

## Germinal Center Derived B Cell Malignancies and Somatic Hypermutation

The B cells malignancies arising later at the germinal center stage of development include diffuse large B cell lymphoma, Hodgkin’s disease and Burkitt’s lymphoma. Each of these have high cure rates with cytotoxic chemotherapy (Sehn et al., 2007; Viviani et al., 2011; Todeschini et al., 2012).

Similarly, to the pro B cells undergoing VDJ recombination, the germinal center B cells are physiologically highly specialized and are undergoing a differing unique genetic event. At this developmental point B cells further alter the DNA sequence and structure of their immunoglobulin genes by undertaking somatic hypermutation of the antibody variable regions. Additionally, the cells also perform class switching in their immunoglobulin heavy chains (Jacob et al., 1991). These processes result primarily from the action of the enzyme activation-induced cytidine deaminase (AID), which deaminates cytidines to uridines within the immunoglobulin gene. These changes are then targeted by DNA repair pathways leading to either point mutations or class switching (Muramatsu et al., 2000; Maul and Gearhart, 2010).

The onset of the activity of this usually highly restricted enzyme is associated with significant genotoxic stress and a lowering of the apoptotic threshold (Zaheen et al., 2009). Similarly, to cells undergoing VDJ recombination the germinal center B cell have a fine balance between negative and positive apoptosis regulators with increased expression of Bax, p53, and c-myc and over expression of the pro-apoptotic receptors including CD95 (Koncz and Hueber, 2012). Overall this balance within the germinal center B cell leads to a programmed apoptotic death unless the B cell is rescued by interaction with antigen or CD40L (Inman and Allday, 2000). As a result, B cells that fail to successfully refine their antibody genes and form functional B cell receptors are destined to die an apoptotic death.

In parallel to the cells undergoing VDJ rearrangement, the B cells in the germinal centers also have dramatic changes to their physiology. Estimates from kinetic studies of germinal center B cells indicate that approximately 50% of these cells die via an apoptotic death every 6 h (Victora et al., 2010) and less than 2% of B cells successfully exit the lymph node (Mayer et al., 2017).

In Hodgkin lymphoma it is apparent that the infection of germinal center B cell with the Epstein Barr virus produces a number of pro-survival mechanisms that allow the infected B cells to resist the normal pro-apoptotic pressures and stimuli that normally remove the large majority of non-functional germinal center B cells (Spender and Inman, 2011). As a result, the Hodgkin lymphoma cells appears to be held suspended in a pro-apoptotic state that is relatively easy to destabilize with additional DNA damage from either cytotoxic drugs or radiation.

In a similar finding to that of ALL where the VDJ recombination process remains on going after the malignant transformation, there is also significant evidence to indicate that B cell malignancies arising from germinal center B cells also have on going activity of aspects of the somatic hypermutation process (Lossos et al., 2000; Pasqualucci et al., 2004; Lenz et al., 2007; Xu-Monette et al., 2019).

### Follicular Lymphoma

The exception to the routine curability of malignancies linked to the somatic hypermutation process in the germinal center is follicular lymphoma. Follicular lymphoma is characteristically highly sensitive to DNA damaging cytotoxic chemotherapy treatment, but despite high response rates and long progression free intervals cure does not occur (Federico et al., 2009; Kahl and Yang, 2016). Whilst the full explanation of this divergence in outcome is awaited, it is likely that the characteristic *t*(14;18)(q32;q21) translocation occurring in follicular lymphoma (Tsujimoto et al., 1985) has a central role on the cells phenotype and apoptotic sensitivity. The impact of this translocation is to greatly enhance expression of the BCL-2 protein which is a powerful inhibitor of the normal apoptotic mechanisms in the germinal center (Huet et al., 2018).

Follicular lymphoma has historically been viewed as having its cell of origin from the germinal center B cell. However, there is increasing evidence that the cells now being identified as the potential follicular lymphoma cell of origin are actually CD27 +ve memory B cells (Sungalee et al., 2014; Tellier et al., 2014). These are long lived cells and it is postulated that they gain the malignant phenotype after repeated transits through the germinal center (Sungalee et al., 2014).

Follicular lymphoma and diffuse large B cell lymphoma (DLBCL) share important similarities with both having a close association with somatic hypermutation and with evidence of on-going somatic hypermutation in the malignant cells (Adam et al., 2007). In contrast the differing timescale and route to malignancy combined with the impact of the presence of BCL-2 activation make the overall biology of these two types of lymphoma very different. Conceptionally the route to malignancy and impact of somatic hypermutation appears to be distinctly different. In DLBCL it is likely that the malignant cells arising during the normal progress through the germinal center with the cells being involved with the full somatic hypermutation process for the first time. In contrast follicular lymphoma is likely to arise from aberrant memory B cells, a cell type that normally has completion somatic hypermutation and no longer expresses AID the key component of this system (Pasqualucci et al., 2004). Memory cells are able to retransit the germinal center and undergo a degree of reactivation of the somatic hypermutation activity (McHeyzer-Williams et al., 2015). However, in the resultant malignant cells the intensity of ongoing somatic hypermutation appears significantly lower in follicular lymphoma than in DLBCL and primary central nervous system lymphoma (Zuckerman et al., 2010).

In addition to the impact of mutation, epigenetic dysregulation plays a key role in the development of follicular lymphoma with mutations occurring in a larger number of key genes controlling changes affecting B cell differentiation and somatic mutation (Kretzmer et al., 2015). The patterns of methylation and hence gene activity differ substantially between follicular lymphoma and high-grade chemotherapy curable germinal center derived lymphomas (Kretzmer et al., 2015).

Whilst it is beyond the scope of this review to examine the details of these changes it is probable that these follicular lymphoma linked changes combined with the high BCL-2 activity may provide a strong counter to the residual impact of the low level activity of the somatic hypermutation associated apoptotic pathways (Zan and Casali, 2015; Huet et al., 2018).

### Physiological Changes in Apoptotic Sensitivity During Somatic Hypermutation

The normal physiology of the germinal center B cell is intricately linked with apoptosis, as a result it is unsurprising that the non-malignant germinal cell B cells are extremely sensitive to DNA damage mediated apoptosis (Lindhout et al., 1995).

The extreme sensitivity of the germinal center to radiation exposure has been documented historically (De Bruyn, 1948; Congdon, 1966; Burge, 1975) and the B cells in the germinal center have been noted to be very sensitive to the induction of DNA damage mediated apoptosis by both cytotoxic drugs and radiation (Thielen and Heinen, 2010).

### Post Somatic Hypermutation B Cell Malignancies

B cells that have successfully completed somatic hypermutation exit from the germinal center and complete the latter stages of maturation into antibody producing plasma cells. In normal healthy B cells at this point the AID activity and SHM phenotype is rapidly lost (Muramatsu et al., 2000; Lenz et al., 2007).

The B cell malignancies that arise from these later stages in development after the completion of both VDJ rearrangement and somatic hypermutation are generally less sensitive to DNA damaging agents and demonstrate no evidence of ongoing SHM activity (Bakkus et al., 1992; Rollett et al., 2006). The diagnoses arising at these points include plasmocytic lymphoma, CLL (mutated variable region) and myeloma which are usually responsive to but are not cured with cytotoxic chemotherapy.

### Overview of the B Cell Malignancies and Their Variation in Chemotherapy Curability

Taken overall, the pattern of chemotherapy curability of malignancies moving along the B cell developmental pathway presents an intriguing picture. The initial malignancies arising closest to the hematopoietic stem cell, have the lowest number of mutations, but only relatively low cure rates with chemotherapy. In contrast the malignancies arising from the next stage in development, that is associated with the VDJ process, generally have more mutations but are highly curable, with over 90% of children with B-ALL being cured with modern therapy. The cells arising from the next stage in B cell development, occurring after VDJ is completed but before somatic hypermutation including Mantle cell lymphoma and chronic lymphocytic leukemia (CLL) are generally sensitive to chemotherapy but non-curable.

The malignancies arising from the next stage in B cell development, during the germinal center phase, include diffuse large B cell lymphoma and Hodgkin’s lymphoma are routinely curable. Finally, the malignancies arising from B cells that have completed VDJ recombination and somatic hypermutation include CLL, plasmocytic lymphoma and myeloma and are non-curable with DNA damaging chemotherapy.

Whilst there are other potential explanations for this unusual biphasic curability curve for B cell malignancies, we would argue that these two peaks of B cell malignancy chemotherapy curability coincide with and are linked to the two apoptosis associated cellular genetic events of immunoglobulin gene VDJ rearrangement and somatic hypermutation/class switching.

As discussed above, the physiology of the B cells during these two processes is dramatically different from any other cells, with extremely rapid turnover, dramatic upregulation of pro-apoptotic pathways and high levels of physiological cell death.

It is apparent that the resultant malignant cells carry some components of the phenotype of the cells that they arise from. We would argue that the presence of these components of the VDJ and somatic hypermutation phenotypes is linked to the extremely high sensitivity of these specific malignancies to DNA damaging chemotherapy.

### T Cell Malignancies

T cell malignancies have a much lower incidence than B cell malignancies despite the numbers of B cells and T cells being similar (Anderson Marjault et al., 1998). T cells have a simpler pathway of genetic recombination to achieve variation in the antigenic recognition of the T cell receptor. The T cell receptor (TCR) alpha chain has only rearrangement of the V and J regions whilst the TCR beta chain has the complete rearrangement of the VDJ components similarly to a B cell (Bassing et al., 2002).

Of the T cell malignancies acute T cell ALL has the highest cure rates with a 75% cure rate in children (Goldberg et al., 2003) but with only 10–15% in adults (Rowe, 2010). Molecular analysis indicates that these cells, similar to B ALL have recombined VDJ genes (van Dongen et al., 2003). Additionally, in a similar fashion to that of B -ALL, it is apparent that the VDJ process can be on going in T-ALL with higher rates of clonal evolution seen in childhood cases compared to adult (Szczepański et al., 2003; Smirnova et al., 2016).

Amongst the malignancies arising later in the T cell developmental pathway ALK +ve anaplastic large cell lymphoma also has a high chemotherapy cure rate with long term survival rates of up to 80% are reported (Gascoyne et al., 1999). ALK +ve large cell lymphoma appears similar to Hodgkin’s lymphoma in that it has undergone V[D]J recombination but failed to produce a functional T cell receptor (Bonzheim et al., 2004). The ALK mutation appears to prevent the natural pattern of apoptosis and holds the cell in a suspended pro-apoptotic state.

The T cell malignancies arising later in the development pathway include peripheral T cell lymphoma, angioimmunoblastic lymphoma and adult T cell leukemia/lymphoma each have a poor prognosis with no significant chemotherapy mediated cure rates (Vose and Armitage, 2008).

### Acute Myeloid Leukemia (AML)

Acute myeloid leukemia has a significant chemotherapy cure rate but in keeping with the experience in B ALL and T ALL the cure rates are significantly higher in children than adults. Overall pediatric AML has a cure rate approaching 70% whilst in adults the cure rate drops from approximately 50% for young adults to only 13% for those aged 60–69 (Shah et al., 2013; de Rooij et al., 2015).

Despite their later development pathways, myeloid precursor cells initially have a high level of activity of the VDJ recombination system. Genetic studies performed in AML indicate that the immunoglobulin heavy chains genes have undergone VDJ rearrangement in 40–50% of cases and that the ongoing expression of the VDJ recombinase associated proteins RAG1 and RAG2 is frequent (Kyoda et al., 1997; Stavroyianni et al., 2003).

In contrast the malignancies arising from mature myeloid cells, have no evidence of VDJ activity and characteristically have modest responses to chemotherapy (Vose and Armitage, 2008; Borcherding et al., 2019).

## Testicular Cancer and Meiosis

Testicular cancer has been curable with chemotherapy for over 60 years (Li et al., 1960) and today patients with metastatic disease have overall cure rates approaching 90% (Feldman et al., 2008).

The malignant cells in testicular cancer usually arise from the pre-malignant precursor carcinoma *in situ* (CIS). This in turn evolves from an abnormal gonocyte, a developmental cell that has failed to mature normally to become a healthy pre-spermatogonia (Sonne et al., 2008, 2009). Recent data indicates that at the onset of testosterone exposure with puberty, the CIS cell, despite not having effectively matured to the a spermatogonium, has some of the key physiological machinery associated with meiosis activated (Jørgensen et al., 2012; Feichtinger and McFarlane, 2019).

In keeping with the thesis of the persistence of unique phenotype within the malignant cells recent data indicates that significant aspects of expression of genes associated with the meiosis phenotype are found in testicular germ cell tumors (Heaney et al., 2012; Bruggeman et al., 2018).

### Physiological Changes in Apoptotic Sensitivity During Meiosis

Meiosis is a complex genetic process that involves genetic recombination and natural apoptosis is an integral component within the process of spermatogenesis. It is estimated that 75% of spermatogenesis is lost via apoptosis (Dunkel et al., 1997).

In the normal process of spermatogenesis, the onset of the process of meiosis has a dramatic effect on the sensitivity to DNA damage induced apoptosis. This has been demonstrated in studies examining the impact of low dose radiation on the survival of cells in the differing stages of spermatogenesis (Rowley et al., 1974; Marjault and Allemand, 2016). The data as shown in Table 3 indicates that the cells that are outside the meiotic process, the quiescent spermatogonial stem cells and the maturing sperm are relatively resistant to induction of apoptosis by low dose radiation despite developing similar levels of DNA damage (Grewenig et al., 2015). In contrast the cells undergoing meiosis, the differentiating spermatogonia and spermatocytes are significantly more sensitive with cell killing rates of up to 99%.

**TABLE 3:** Variation in the sensitivity to the induction of apoptosis with DNA damaging radiation and cytotoxic chemotherapy during spermatogenesis.

Cell	Genetic Event	Radiation 1 Gy	ID 50 mg/kg		
		% Survival	Cyclophosphamide	Cytarabine	Doxorubicin
Spermatogonial stem cell	Mitosis	50%	>200	>7000	3
Spermatogonial cell differentiating	Meiosis	1%	30	16	1
Spermatocyte (Leptotene)	Meiosis	1%	–	–	–
Spermatocyte (Pachytene)	Meiosis	50%	–	–	–
Spermatid	Maturation	52%	–	–	–

*Radiation data (Rowley et al., 1974). ID 50 data (Lu and Meistrich, 1979). Doxorubicin data.*

Experimental data in murine models examining the impact of a number of cytotoxic chemotherapy drugs on apoptosis in spermatogenesis also indicates dramatic changes in sensitivity to chemotherapy between spermatogonial stem cells and differentiating spermatogonial cells (Lu and Meistrich, 1979). As shown in Table 3 the difference in ID50 concentration can vary up to more than a 100-fold difference which is far greater than the ratios seen in conventional malignancies (Izumi et al., 2017).

It is apparent that the processes involved in meiosis are closely linked to naturally occurring apoptosis in health. We would argue that in testicular cancer the malignant cells that arise from these abnormal parent cells that have partially activated components of the meiotic phenotype. As a result, they maintain some of the unique physiology and heightened apoptotic sensitivity that naturally occurs at this unique biological point.

## Gestational Malignancies and Nuclear Fusion

The gestational malignancies are rare but have been curable with chemotherapy since the 1950s (Hertz et al., 1956). The majority of cases happen after a complete molar pregnancy and in this situation a cure rate of 100%, with the use of often low dose single agent chemotherapy, can be expected (Sita-Lumsden et al., 2012). The clinically more challenging forms of gestational malignancies, choriocarcinoma and placental site trophoblast tumor generally arise from otherwise genetically normal pregnancies and have high cure rates with combination chemotherapy (Schmid et al., 2009; Alifrangis et al., 2013).

Whilst there is no normal counterpart to the cells of a molar pregnancy, it is apparent that it shares considerable phenotypic characteristic with an early healthy trophoblast cell. In contrast the cells of choriocarcinoma and PSST have phenotypic and epigenetic similarities with more slightly more developmentally mature cells (Kurman et al., 1984; Savage et al., 2019). Similar to the situation in the B cell malignancies it appears that the malignant trophoblast cells remain frozen with their developmental phenotype and do not follow the standard maturation and developmental pathways of trophoblast/placental cells (Savage et al., 2019).

The timing of the developmental origin of the gestational trophoblast tumors appears to have a close relationship with the chemotherapy sensitivity of the malignancies arising along this route. Molar pregnancies that arise at the time of fertilization have a cure rate approaching 100% and patients with this diagnosis generally only require low dose single agent chemotherapy (Sita-Lumsden et al., 2012). Gestational choriocarcinoma which arises slightly later in developmental has chemotherapy cure rates approaching 95% but with most patients requiring more intensive combination chemotherapy (Alifrangis et al., 2013). The rarer diagnosis PSST which is believed to arise later in trophoblast cell development has a lower overall cure rate for patients with metastatic disease of approximately 50% (Schmid et al., 2009).

### Physiological Changes in Apoptotic Sensitivity Following Nuclear Fusion

In health gametes and the cells of conception demonstrate a change in their sensitivity to the induction of DNA damage mediated apoptosis in a relatively short period after fertilization. Clinical data indicate that sperm are relatively resistant to DNA damage, with sperm counts in cancer patients only subsiding significantly 1–2 months after the commencement of cytotoxic chemotherapy (Meistrich et al., 1997). This resistance to the induction of DNA damage mediated apoptosis is also supported by data shown in Table 3 that indicates that radiotherapy (at 1Gy) only causes a 50% reduction in spermatid counts compared to a 99% reduction in the number of early spermatocytes (Rowley et al., 1974).

There is little data on the sensitivity of human ova and the early cells of conception to DNA damaging cytotoxic drugs. However, *in vitro* animal data suggests that there is an appreciable difference in sensitivity to methotrexate mediated apoptosis between the newly fertilized ova and the early stages of the developing blastocyst. Murine data looking at the impact of methotrexate exposure on maturing ova indicates that concentrations of methotrexate of 20uM and above are clearly cytotoxic (Takai et al., 2007). However, the cells are able to tolerate exposure to methotrexate at 10 μM with only modest reductions in the speed of germinal vesicle breakdown and polar body extrusion but without an impact on cell viability (Tian et al., 2018).

In fertilized zygotes, in both murine and bovine *in vitro* models, a biphasic impact of methotrexate exposure is seen. Fertilized ova and zygotes up to 8 cell stage were largely resistant to exposure to methotrexate at 10 μM. In contrast, methotrexate at 10 μM resulted in the total loss of viability for 8 cells zygotes in the bovine system (Kwong et al., 2010) and a major reduction in viability in the mouse model (O’Neill, 1998). The reason for this increasing sensitivity to methotrexate induced apoptosis is unclear. It is apparent that the cells are increasing their thymidine requirements (O’Neill, 1998) at this stage but also making profound and rapid changes to their patterns of gene expression (Fraser and Lin, 2016).

The *in vitro* documentation of extreme sensitivity of the early cells of conception to cytotoxic drugs is also reflected clinically in the management of ectopic pregnancies where a single dose of 50 mg of methotrexate is employed (Creinin et al., 1996).

The very high degree of sensitivity to cytotoxic drugs of the cells after conception occurs for only a relatively short time and as the placenta matures it undergoes a rapid change in sensitivity to DNA damage induced apoptosis. This process that is complete by the end of the first trimester by when the trophoblast cells (Sand et al., 1986) and the placenta are resistant to cytotoxic drug induced apoptosis (Abellar et al., 2009). Of the note the very rare proliferative conditions that arise from the mature cells of the placental exhibit a high degree of resistance to chemotherapy drugs (Farasatinasab et al., 2016).

At present there is little data on the underlying mechanisms for the high degree of chemotherapy sensitivity for both the native and malignant trophoblast cells. Recent whole genome sequencing and epigenetic analysis suggests that gestational trophoblast tumors do not have any significant mutational burden and are likely to arise during aberrations of methylation during the early stages of placental development (Rowley et al., 1974; Xing et al., 2019). Following fertilization there are dramatic changes in the methylation patterns of the trophoblast cell (Fraser and Lin, 2016) and it is likely that these changes in gene expression and DNA conformation are the key drivers in the rapidly changing sensitivity to induction of apoptosis from cytotoxic chemotherapy induced DNA damage.

### Childhood Malignancies and Gastrulation

At present there is considerable debate regarding the origin of the rare childhood malignancies. Previously we and others have suggested that these rare malignancies with their very primitive pathology may be linked to defects in gastrulation (Ewing, 1921; von Levetzow et al., 2011; Savage, 2015). We hypothesize that these cells remain trapped in a normally transient phenotype in similar way the B cell malignancies are trapped at the point of origin cell type.

The curability of these malignancies with cytotoxic chemotherapy was originally seen nearly 50 years ago and current cure rates approach 70% for neuroblastoma, 75% for Ewing sarcoma 74 and 90% for Wilms tumor (Smith et al., 2010).

### Physiological Changes in Apoptotic Sensitivity During Gastrulation

**FIGURE 3:**
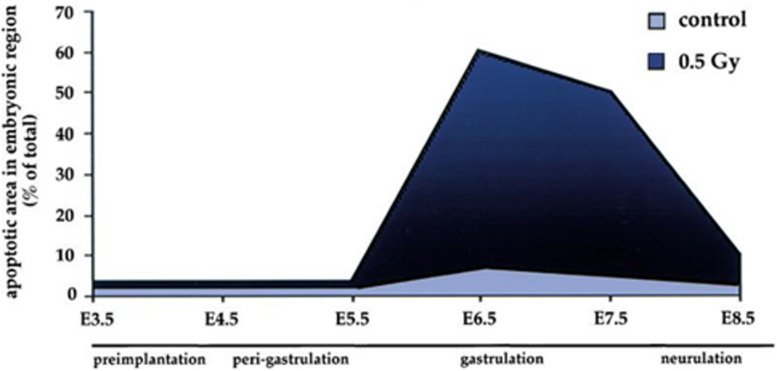
Sensitivity of embryonic cells to low dose radiation (0.5 Gy) before, during and after gastrulation. Data and graph taken from Heyer et al. (2000).

Whilst gastrulation does not include DNA mutation or recombination the process has a major impact on apoptotic sensitivity. In a murine model, the dramatic changes in the sensitivity of cells to DNA damage associated with gastrulation are demonstrated in Figure 3. An exposure of 0.5Gy resulted in <5% apoptotic embryonic cell death at day 5 just prior to gastrulation. In contrast at day 6.5 the apoptotic cell death response to the same low dose of radiation rose to 60%. By day 8.5 as the gastrulation process was completing the apoptotic response fell back to 10% (Heyer et al., 2000).

The relationship to defects in gastrulation and the etiology of the rare childhood malignancies remains an area of debate. However, it is apparent that in a similar way to the impact of VDJ recombination, meiosis and nuclear fusion the process of gastrulation is closely related to dramatic changes in apoptotic sensitivity.

## Differential Chemotherapy Sensitivity in Non-Mutational and Single Mutation Malignancies

Classically the paradigm for oncogenesis is centered on the sequential development of mutation leading to the development of the malignant phenotype. This appears to be the case for the majority of solid cancers (Fearon and Vogelstein, 1990) and also for many lymphoid malignancies (Greaves et al., 2003).

A small number of relatively rare malignancies, each of which has a short developmental time for oncogenesis, appear to arise via alternate routes. Recent whole genome sequencing and epigenetic analyses have provided an insight into these differing routes to oncogenesis and also potential factors affecting chemotherapy sensitivity and chemotherapy curability.

### Non-mutational Malignancies

Two rare malignancies appear to have their route to oncogenesis occurring from epigenetic changes alone without any documented mutational drivers.

In the childhood CNS malignancy CIMP ependymoma, a series of 47 cases were analyzed by whole genome analysis, with the results indicating a very low mutational burden and an absence of documented oncogene or driver mutations (Mack et al., 2014). In contrast to the absence of mutation, epigenetic analysis indicated consistent abnormalities of methylation impacting on the action of the Polycomb repressive complex (Mack et al., 2014).

More recently we have reported the first case of gestational choriocarcinoma to be analyzed with whole genome sequencing and full methylation analysis (Savage et al., 2019). Similarly, to the findings in CIMP ependymoma this cancer also has no appreciable mutational burden. However, the cells appear to have a defect in the natural progression of methylation that leaves the malignant cell fixed with a persisting phenotype of an early trophoblast cell.

Whilst the exact timing of the onset of oncogenesis in gestational choriocarcinoma is yet to be determined it is likely that the malignant cells arise from cells within the first 2 weeks after conception and nuclear fusion. As discussed previously normal healthy cells at this point are immensely sensitive to cytotoxic chemotherapy *in vitro* (Kwong et al., 2010) and *in vivo* as demonstrated by the efficacy of low dose methotrexate in the treatment of ectopic pregnancies (Creinin et al., 1996).

Whilst these two malignancies have similar etiology, they have dramatically different responses to chemotherapy treatment as shown in Table 4. CIMP ependymoma is characteristically highly resistant to cytotoxic chemotherapy treatment and carries an extremely poor prognosis (Bouffet and Foreman, 1999). In contrast gestational choriocarcinoma is immensely sensitive to cytotoxic chemotherapy treatment and has a 95% cure rate (Alifrangis et al., 2013).

**TABLE 4:** Comparison of the route to oncogenesis, relationship to unique genetic events and chemotherapy curability for non-mutational and single mutation malignancies.

	Non-mutational malignancies		Single mutational malignancies	
Diagnosis	Gestational Choriocarcinoma	Ependymoma	Infantile ALL	Pediatric rhabdoid cancer
Mutations	Nil	Nil	MLL	SMARCB1
Oncogenesis	Defect in Methylation	Defect in Methylation	Mutational	Mutational
Genetic Event	Nuclear Fusion	Nil	VDJ Recombination	Nil
Chemotherapy Response Rate	100%	11%	95%	50%
Chemotherapy Cure Rate	95%	0%	40%	<10%

As discussed above we would argue that the key difference that affects the response to chemotherapy, for these two non-mutational malignancies, is the persistence of the ongoing phenotype of the early post nuclear fusion trophoblast cell in choriocarcinoma.

### Single Mutational Malignancies

Highly divergent sensitivity to DNA damaging chemotherapy is also seen in two other rare malignancies which are each characterized by a single mutation.

Pediatric rhabdoid malignancy has a single driver mutation which is of SMARCB1 tumor suppressor gene (Kieran et al., 2012). Similarly, infantile ALL also has a single driver mutation in the MLL gene (Dobbins et al., 2013).

Aside from these mutations both infantile ALL and the pediatric rhabdoid malignancies are otherwise mutationally bland with a negligible level of mutation (Kieran et al., 2012; Dobbins et al., 2013).

Despite this apparent similarity of genetic structure and route to oncogenesis these two malignancies also differ dramatically in their sensitivity to DNA damaging chemotherapy treatment. Pediatric rhabdoid malignancies are characterized by a very high level of resistance to DNA damaging cytotoxic chemotherapy. The prognosis for this rare cancer is very poor with the median survival of patients with metastatic disease of less than 1 year (Reinhard et al., 2008; Brennan et al., 2016). In contrast infantile ALL has a high response rate to chemotherapy and approximately 40% of patients are cured (Pieters et al., 2007).

However, whilst the two malignancies are both mutationally bland aside from their single driver mutation there is key difference between them. Infantile ALL arises in cells that are in the earliest stages of VDJ rearrangement that have either germline or an incompletely rearranged VDJ regions (Jansen et al., 2007). In keeping with cases of ALL arising slightly later in the development pathway infantile ALL cells have on going significant expression of the RAG1/2 enzymes indicating that significant components of the early VDJ phenotype are retained in the malignant cells (Jansen et al., 2007).

From these four rare malignancies we can see the extremes of sensitivity to chemotherapy treatment that does not appear to be influenced by the absence of mutation, or by the route to oncogenesis being methylation changes. However, for the two chemotherapy curable malignancies it is apparent that they are closely linked, via their parent cell, to the genetic events of either VDJ recombination or nuclear fusion. We would argue that it is the partial persistence of the unique phenotype associated with these events that leads the cells to retain extremely high levels of sensitivity to DNA damaging therapies.

## Discussion

A number of malignancies have been routinely curable with chemotherapy treatment for over 60 years. Review of the natural history of these cells indicates that they each arises from a special transient cell type that is intricately involved with one of the key physiological processes of immunoglobulin gene rearrangements, meiosis, nuclear fusion or gastrulation.

The chemotherapy sensitivity of these malignancies appears to mirror the extreme natural sensitivity to the induction of apoptosis that these parent cells naturally pass through as part of these transient processes in health.

This interpretation of the biological and clinical data offers an explanation as to why other malignancies have not become curable despite immense research endeavors.

At present there is little data on the detailed mechanisms as to how these dramatic changes in apoptotic sensitivity occur. With more information it may be possible to devise therapeutic approaches that could therapeutically exploit these dramatic biological effects.

## Data Availability Statement

All datasets generated for this study are included in the article/supplementary material/reference list.

## Author Contributions

PS wholly designed, researched the study and wrote the manuscript.

## Conflict of Interest

The author declares that the research was conducted in the absence of any commercial or financial relationships that could be construed as a potential conflict of interest.
